# Investigating the Role of Easter Island in Migration of Zika Virus from South Pacific to Americas

**DOI:** 10.3201/eid2411.180586

**Published:** 2018-11

**Authors:** Edson Delatorre, Jorge Fernández, Gonzalo Bello

**Affiliations:** Instituto Oswaldo Cruz/Fiocruz, Rio de Janeiro, Brazil (E. Delatorre, G. Bello);; Public Health Institute of Chile, Santiago, Chile (J. Fernández)

**Keywords:** Zika virus, epidemics, phylogeny, Pacific Islands, Americas, viruses, vector-borne infections, mosquitoborne, South Pacific, Easter Island, migration, French Polynesia

## Abstract

The role of Easter Island in the dissemination of Zika virus from the Pacific islands into the Americas remains unclear. We analyzed new Zika virus sequences from Eastern Island and found that Zika virus was independently disseminated from French Polynesia into the Americas and Easter Island at around the same time.

Zika virus is a mosquitoborne flavivirus associated with several recent outbreaks in human populations in the Pacific region and the Americas. Phylogeographic studies indicate that Zika virus strains circulating in South Pacific islands and Latin America comprise a single lineage (ZIKV_SP-AM_) that arose because of sequential single viral disseminations from Southeast Asia into French Polynesia and from the South Pacific into Latin America (*1*–*5*). However, whether the ancestral Zika virus strain introduced into the Americas arose directly from French Polynesia or from another South Pacific island is unclear.

In early 2014, a Zika virus outbreak occurred in Easter Island (*6*), months before the first identification of Zika virus in Brazil (*7*). Easter Island is located at the southeastern edge of the Polynesian Triangle, roughly equidistant from French Polynesia and the South America mainland. Geographic position and intense touristic activity make Easter Island a potential staging post in the spread of Zika virus from French Polynesia to continental America. This hypothesis was suggested previously (*4*), but the study used a limited sequence dataset. We tested this hypothesis by using a more comprehensive dataset of Zika virus sequences from the South Pacific.

Blood samples from suspected Zika virus–infected human patients who visited the emergency unit of Hanga Roa Hospital on Easter Island during January–May 2014 were sent to the Public Health Institute of Chile for characterization, according to Ministry of Health of Chile guidelines for surveillance of transmissible diseases. The complete E and partial NS5 genes of 7 Zika virus strains were obtained as previously described (*6*). The concatenated fragments were aligned with Zika virus Asian genotype sequences available in GenBank and used for spatiotemporal viral diffusion reconstruction (Technical Appendix).

The overall timescale of the phylogenetic tree estimated for Zika virus Asian genotype strains was fully consistent with previous reports (*1**–**5*; Technical Appendix Table 2). Asymmetric (Technical Appendix Figure 1) and symmetric (Technical Appendix Figure 2) phylogeographic models placed the most recent common ancestor of the Asian genotype epidemic strains in Southeast Asia (posterior state probability [PSP] >0.96) at 1999 (Bayesian credible interval [BCI] 1996–2002) and support 2 independent disseminations from Southeast Asia into the Pacific region—the first in Micronesia around 2007, with no further spread, and the second originating the ZIKV_SP-AM_ lineage that fueled epidemics during 2013–2016 in the South Pacific and the Americas.

The origin of the ZIKV_SP-AM_ lineage was traced to French Polynesia (PSP >0.98) at 2013.3 (BCI 2012.9–2013.6). This lineage exhibits strong geographic subdivision; several highly supported island-specific monophyletic subclades nested among basal strains from French Polynesia (*5*), besides the ZIKV_AM_ subclade (Technical Appendix Figures 1, 2). We found 2 major clades within the ZIKV_SP-AM_; clade I (posterior probability [PP] 0.97) contains strains from French Polynesia, the Americas, New Caledonia, and Vanuatu, whereas clade II (PP >0.94) encloses strains from Easter Island and the Cook Islands. Zika virus strains from Fiji, American Samoa, Samoa, Tonga, and the Solomon Islands branched together in a large but not well-supported (PP <0.57) monophyletic group.

French Polynesia was the most probable source location of the Zika virus clade I strains introduced into the Americas (PSP 1), Vanuatu (PSP 1), and New Caledonia (PSP >0.98), as well as of the clade II strain introduced into Easter Island (PSP >0.77), refuting the hypothesis of introduction of Zika virus into the Americas through Easter Island. The source location of the clade II strain of the Cook Islands was traced to Easter Island (PSP >0.34) or Easter Island (PSP >0.37). Our analyses indicate that Zika virus was introduced into Easter Island, New Caledonia, the Cook Islands, and the Americas at around the same time (BCI 2013.6–2014.8), supporting a much longer period of undetected Zika virus transmission in the Americas (≈15 months) (*7*,*8*) than in those South Pacific islands (<1 month) (*8*). Zika virus also spread from French Polynesia into Samoa, Fiji, Tonga, and American Samoa from late 2015 to early 2016, probably following a stepping-stone process (Technical Appendix Figures 1, 2). Migratory routes between those islands, however, were difficult to resolve (inconsistency between phylogeographic models) or do not fully agree with epidemiologic data (e.g., the dissemination from Tonga to Fiji) (*8*).

Our results indicate that French Polynesia was the main hub of dissemination of the ZIKV_SP-AM_ lineage and seeded independent outbreaks in several South Pacific islands (including Easter Island) and the Americas from late 2013 to mid-2014, coinciding with a peak in the number of suspected Zika cases in French Polynesia. The long period of cryptic circulation of Zika virus in the Americas, the early detection of Zika virus in the Caribbean in December 2014 (*4*), and the reported dissemination of dengue (*9*) and chikungunya (*10*) viruses from French Caribbean territories into French Polynesia during 2013–2014 support the hypothesis that Zika virus might have been introduced to and circulated in the Caribbean region for several months before its detection in Brazil in 2015. Human movement between overseas French territories might create an epidemiologic link for arboviral transmissions between the South Pacific and the Caribbean region.

Technical Appendix Geographic dissemination of Zika virus Asian genotype throughout South Pacific and the Americas, estimated with asymmetric and symmetric models.
