# Supplementary material for: Investigating the Role of Easter Island in Migration of Zika Virus from South Pacific to Americas
Source: Emerg Infect Dis. 2018 Nov;24(11):2119–21. doi: 10.3201/eid2411.180586 (PMC6200010; doi:10.3201/eid2411.180586)
Supplement: Technical Appendix — Geographic dissemination of Zika virus Asian genotype throughout South Pacific and the Americas, estimated with asymmetric and symmetric models. [file 18-0586-Techapp-s1.pdf]

Article DOI: https://doi.org/10.3201/eid2411.180586

Investigating the Role of Easter Island in Migration of Zika Virus from South Pacific to Americas

Technical Appendix

Methods

Zika Virus Sequencing

Blood samples were collected from symptomatic patients following the epidemiologic surveillance guidelines proposed by the Ministry of Health of Chile (Documents B51/833–2015 and 158/04 for surveillance of transmissible disease). These guidelines include the obligatory notification of suspected cases and require the sending of samples to the Public Health Institute of Chile for diagnostic and characterization of viral agents.

Viral RNA was extracted from serum using an easyMAG extraction system (bioMerieux) and the complete E and partial NS5 genes were amplified by nested RT-PCR in two fragments (PCR primers are described in Technical Appendix Table 1) and sequenced bidirectionally by using Sanger Sequencing, as previously described (*2*). Nucleotide sequences were assembled and edited using the SeqMan program (DNASTAR, Madison, WI).

Sequence Dataset and Phylogeographic Analysis

Concatenated E and NS5 fragments of seven Easter Island ZIKV strains were aligned with ZIKV Asian genotype complete coding sequences (CDS) and E sequences available on GenBank by December 2017 with information about country of infection and date of isolation from: Southeast Asia (CDS sequences), Pacific Islands (CDS and E sequences) and the Americas (CDS sequences) (alignment available upon request). Representative subsets of sequences from the Americas and Singapore were selected using a previously described strategy (*3*).

The spatiotemporal viral diffusion pattern was reconstructed using the Markov chain Monte Carlo (MCMC) algorithm implemented in the BEAST v1.8 package (*4*) with a relaxed uncorrelated lognormal molecular clock model, the GTR+Γ4 nucleotide substitution model, a Bayesian Skyline tree coalescent model and both reversible (symmetric) and nonreversible (asymmetric) discrete phylogeographic models. MCMC were run sufficiently long (100 million MCMC steps) to ensure stationary and convergence of all parameters (Effective Sample Size >200), through inspection with Tracer v1.6 (http://tree.bio.ed.ac.uk/software/tracer/). After discarding the 10% burn-in, Maximum Clade Credibility (MCC) trees were generated with TreeAnnotator (BEAST v1.8) and visualized with FigTree v1.4 (http://tree.bio.ed.ac.uk/software/figtree/). Results are presented in Technical Appendix Table 2.

References

1. Quick J, Grubaugh ND, Pullan ST, Claro IM, Smith AD, Gangavarapu K, et al. Multiplex PCR method for MinION and Illumina sequencing of Zika and other virus genomes directly from clinical samples. Nat Protoc. 2017;12:1261–76. PubMed http://dx.doi.org/10.1038/nprot.2017.066

2. Tognarelli J, Ulloa S, Villagra E, Lagos J, Aguayo C, Fasce R, et al. A report on the outbreak of Zika virus on Easter Island, South Pacific, 2014. Arch Virol. 2016;161:665–8. PubMed http://dx.doi.org/10.1007/s00705-015-2695-5

3. Delatorre E, Mir D, Bello G. Tracing the origin of the NS1 A188V substitution responsible for recent enhancement of Zika virus Asian genotype infectivity. Mem Inst Oswaldo Cruz. 2017;112:793–5. PubMed http://dx.doi.org/10.1590/0074-02760170299

4. Drummond AJ, Rambaut A. BEAST: Bayesian evolutionary analysis by sampling trees. BMC Evol Biol. 2007;7:214. PubMed http://dx.doi.org/10.1186/1471-2148-7-214

5. Faria NR, Azevedo RDSDS, Kraemer MUG, Souza R, Cunha MS, Hill SC, et al. Zika virus in the Americas: early epidemiological and genetic findings. Science. 2016;352:345–9. PubMed http://dx.doi.org/10.1126/science.aaf5036

6. Faria NR, Quick J, Claro IM, Thézé J, de Jesus JG, Giovanetti M, et al. Establishment and cryptic transmission of Zika virus in Brazil and the Americas. Nature. 2017;546:406–10. PubMed http://dx.doi.org/10.1038/nature22401

7. Metsky HC, Matranga CB, Wohl S, Schaffner SF, Freije CA, Winnicki SM, et al. Zika virus evolution and spread in the Americas. Nature. 2017;546:411–5. PubMed http://dx.doi.org/10.1038/nature22402

8. Pettersson JH, Eldholm V, Seligman SJ, Lundkvist Å, Falconar AK, Gaunt MW, et al. How did Zika virus emerge in the Pacific Islands and Latin America? MBio. 2016;7:e01239-16. PubMed http://dx.doi.org/10.1128/mBio.01239-16

Technical Appendix Table 1. Primers used in this study

Target	Reference	Start pos	Stop pos	Size (bp)	Primer name	Sequence (5′ → 3′)
NS5	KJ776791	8167	8189	1040	400_28_out_L*	GGTGGGGGATTGGCTTGAAAAA
		9184	9206		400_30_out_R*	TAATCCCAGCCCTTCAACACCA
E Fragment 1		979	1000	416	400_4_out_L*	TCAGGTGCATAGGAGTCAGCAA
		1415	1394		400_4_out_R*	GGAGCCATGAACTGACAGCATT
E Fragment 2		1257	1278	731	400_5_out_L*	AGAACGTTAGTGGACAGAGGCT
		1966	1987		400_6_out_R*	CCATCTGTCCCTGCGTACTGTA
E Fragment 3		1876	1897	668	400_7_out_L*	TGAAGGGCGTGTCATACTCCTT
		2524	2543		E3_out_R	CCCTGTACCGCATCTCGTCT

*Primers described in (*1*).

Technical Appendix Table 2. Posterior estimates of evolutionary parameters of ZIKV Asian genotype

Study	Phylogeographic model	Evolutionary rate (95% BCI)	TMRCA (95% BCI) Pandemic Asian clade	Southern Pacific clade	American clade
Faria et al. 2016 (*5*)	–	1.1×10^{-3} (0.9×10^{-3}–1.3×10^{-3})	–	–	2014.0 (2013.6–2014.3)
Faria et al. 2017 (*6*)	Asymmetric	1.1×10^{-3} (1.0×10^{-3}–1.3×10^{-3})	–	–	2014.2 (2013.9–2014.4)
Metsky et al. 2017 (*7*)	–	1.2×10^{-3} (1.0×10^{-3}–1.3×10^{-3})	–	–	2014.1 (2013.6–2014.6)
Pettersson et al. 2016 (*8*)	–	1.2×10^{-3} (1.1×10^{-3}–1.3×10^{-3})	2001.7 (2000.6–2002.7)	2013.3 (2012.8–2013.4)	2013.9 (2013.8–2014.2)
This study	Symmetric	0.9×10^{-3} (0.8×10^{-3}–1.1×10^{-3})	1999.5 (1996.4–2002.2)	2013.3 (2012.9–2013.6)	2013.9 (2013.6–2014.1)
	Asymmetric	0.9×10^{-3} (0.8×10^{-3}–1.1×10^{-3})	1999.4 (1996.3–2002.4)	2013.3 (2012.8–2013.6)	2013.9 (2013.6–2014.1)

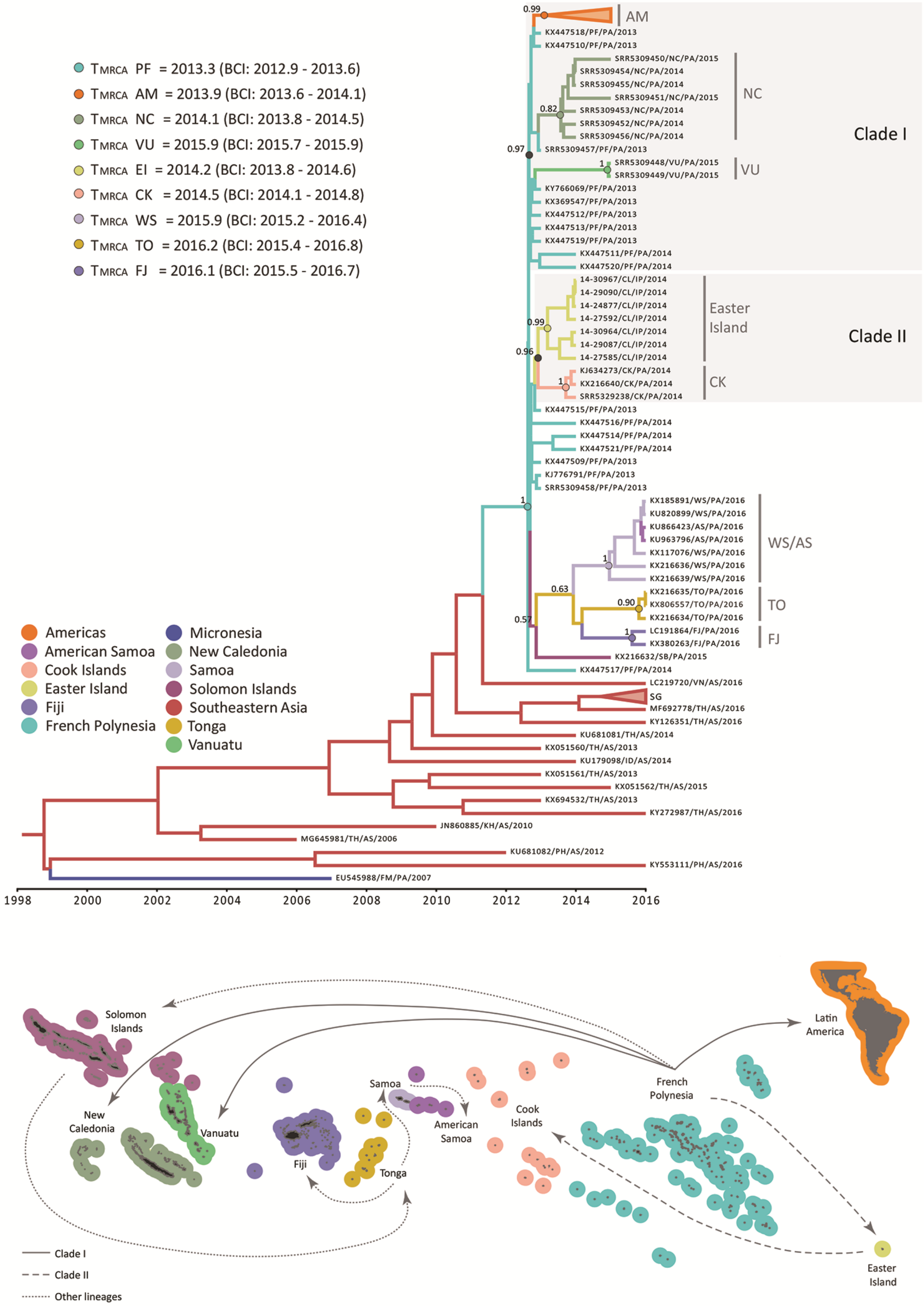

Technical Appendix Figure 1. Geographic dissemination of Zika virus Asian genotype, South Pacific and the Americas. Bayesian time-scaled maximum clade credibility phylogeny estimated from 110 Zika virus Asian genotype sequences with the asymmetric model. The branches' colors represent the most

probable location state (geographic region) of their ancestral node. The times to the most recent common ancestor of key nodes (colored circles) are indicated. Numbers at selected nodes indicate the clade posterior probabilities. The shaded areas highlight the Zika virus clades we have described. All horizontal branch lengths are drawn to a scale of years. Arrows between locations represent the estimated viral migration events and its line’s pattern discriminate the viral flux of each Zika virus clade. The sequences of the Eastern Island isolates were submitted to GenBank under accession numbers MG982560–MG982573. AM, Americas; AS, American Samoa; CK, Cook Islands; EI, Easter Island; FJ, Fiji; NC, New Caledonia; PF, French Polynesia; TMRCA, time to the most recent common ancestor; TO, Tonga; VU, Vanuatu; WS, Samoa.

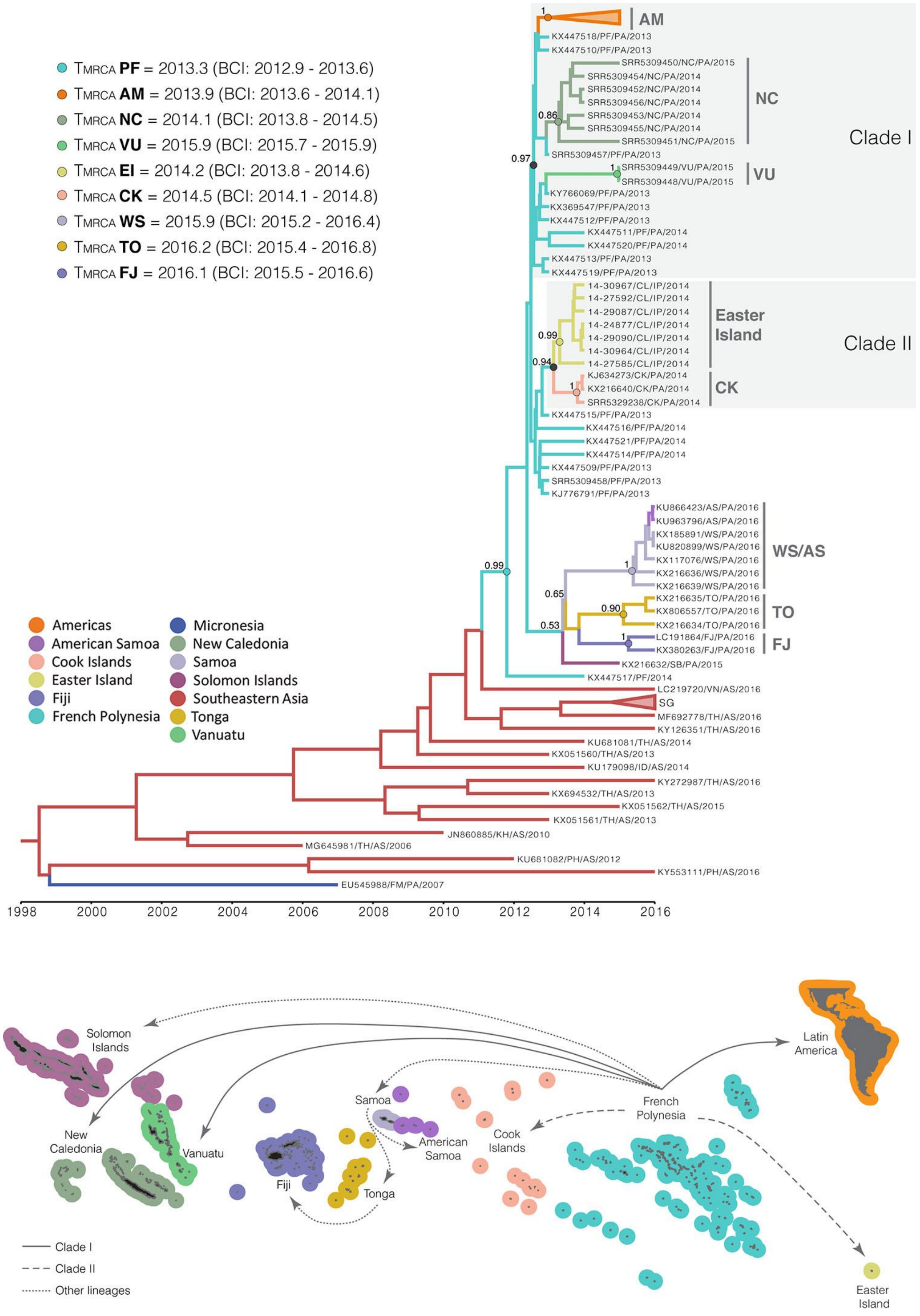

Technical Appendix Figure 2. Geographic dissemination of Zika virus Asian genotype, South Pacific and the Americas. Bayesian time-scaled maximum clade credibility phylogeny estimated from 110 Zika virus Asian genotype sequences with the symmetric model. The branches' colors represent the most

probable location state (geographic region) of their ancestral node. The times to the most recent common ancestor of key nodes (colored circles) are indicated. Numbers at selected nodes indicate the clade posterior probabilities. The shaded areas highlight the Zika virus clades we have described. All horizontal branch lengths are drawn to a scale of years. Arrows between locations represent the estimated viral migration events and its line’s pattern discriminate the viral flux of each Zika virus clade. The sequences of the Eastern Island isolates were submitted to GenBank under accession numbers MG982560–MG982573. AM, Americas; AS, American Samoa; CK, Cook Islands; EI, Easter Island; FJ, Fiji; NC, New Caledonia; PF, French Polynesia; TO, Tonga; VU, Vanuatu; WS, Samoa; TMRCA, time to the most recent common ancestor.
